# Global Prevalence and Burden of Orofacial Clefts: A Systematic Analysis for The Global Burden of Disease Study 2019

**DOI:** 10.1097/SCS.0000000000009591

**Published:** 2023-08-15

**Authors:** Rami S. Kantar, Usama S. Hamdan, John N. Muller, Kshipra Hemal, Robert A. Younan, Mario Haddad, Antonio M. Melhem, J. Peter W. Don Griot, Corstiaan C. Breugem, Ali H. Mokdad

**Affiliations:** 1 Global Smile Foundation, Norwood, MA, USA; 2 The Hansjorg Wyss Department of Plastic Surgery, NYU Langone Health, New York City, NY, USA; 3 Department of Plastic and Reconstructive Surgery, Amsterdam University Medical Center, Amsterdam, Netherlands; 4 Institute for Health Metrics and Evaluation, University of Washington, Seattle, WA, USA; Department of Health Metrics Sciences, University of Washington, Seattle, WA, USA.

**Keywords:** Cleft, Cleft Care, Cleft Lip, Cleft Palate, Orofacial Clefts, Oral Health, Global Health, Global Surgery

## Abstract

Orofacial clefts, in particular cleft lip and cleft palate, are among the most common congenital anomalies. Despite guidelines recommending early surgical correction, a global backlog of untreated patients persists. This has made orofacial clefts an attractive target for global cleft care initiatives. The most recent global burden of orofacial clefts was estimated to be 529,758.92 DALYs (95% UI: 362,492.88 – 798,419.69 DALYs), while the global prevalence of orofacial clefts was estimated to be 4.6 million (95% UI: 3.8 – 5.7 million). An inverse relationship exists between sociodemographic index and burden of orofacial clefts. Sub-Saharan Africa, Middle East/North Africa, and South Asia are the regions carrying the most significant burden of orofacial clefts. This manuscript provides updated estimates of the global burden and prevalence of orofacial clefts, acting as a guide to direct future investments, resources, and initiatives from individuals and organizations engaged in global cleft care delivery with the goal of building sustainable cleft care capacity where it is needed the most.

## Introduction

Congenital orofacial clefts are among the most common congenital anomalies.^1^ Guidelines suggest that for patients with these anomalies, the cleft lip should ideally be repaired within the first year of life, while clefts of the palate, if present, should be corrected by 18 months of age.^1^ If untreated, these anomalies have been associated with increased morbidity and mortality.^2^ Nevertheless, a significant backlog of untreated patients with variable geographical distribution persists.^3^

This significant number of untreated patients has made orofacial cleft care in low to middle-income countries a target for international clinical initiatives.^4^ These international cleft care initiatives have meaningfully contributed to alleviating the burden associated with orofacial clefts, and have varied widely in breadth of clinical care, models of care delivery, sustainability and geographical distribution.^4^

A recent study has demonstrated that there is a persistent strong negative association between the size of the global surgical workforce and burden of orofacial clefts, suggesting that reinforcing the global surgical workforce will assist in alleviating the global burden of orofacial clefts.^3^ In this study, we utilize updated Global Burden of Disease data to model and highlight the global prevalence and burden rates of orofacial clefts. We aspire that this updated manuscript serves as a guide for future global cleft care initiatives. By estimating and geographically mapping the burden and prevalence of congenital orofacial clefts, initiatives may be able to specifically target those in most need.

## Methods

### Overview

The Global Burden of Disease (GBD) Study spearheaded by the Institute for Health Metrics and Evaluation (IHME) is the most comprehensive study evaluating the trends, distribution, and impact of diseases and injuries around the world.^5^ In the most recent update to the study, GBD 2019, the prevalence, trends, and impact of 369 diseases and injuries in 204 countries and territories are systematically analyzed.^5^ The detailed methodology of GBD 2019 has been previously published and extensively described elsewhere.^5^

### Measures of Interest

In this study, the measures of interest include disability-adjusted life years (DALYs) as a surrogate for orofacial clefts burden, and prevalence. DALYs are computed through the sum of Years of Life Lost (YLLs) and Years of Healthy Life Lost due to Disability (YLDs).^5^ YLDs are computed as a product of the disability weight, ranging from 0 for perfect health to 1 for death, and the prevalence of a disease.^5^ The disability weights associated with orofacial clefts have been previously reported.^3,5^ Orofacial cleft burden and prevalence are reported per 100,000 in this paper. All measures are reported with uncertainty intervals (UI) ranging from 2.5% to 97.5%.^5^

### Case Definition

In the GBD 2019 study, orofacial clefts include isolated cleft lip or palate, and combined cleft lip and palate.^5^ Craniofacial clefts that do not include the oropharynx are not included in this disease definition.^5^ The GBD 2019 study also considers dysmorphic characteristics and treatment sequelae as well as includes corresponding ICD-10 codes.^5^

### Socio-Demographic Index Analysis

The GBD 2019 study utilized Socio-Demographic Index (SDI) as an indicator of economic and social conditions affecting health outcomes in different locations.^5^ SDI represents the geometric mean of 0 to 1 indices of total fertility rate for individuals younger than 25 years, mean education for those 15 years and older, and lag-distributed income per capita.^5^

### Geographical Distribution

Global rates of orofacial cleft burden and prevalence are reported. Burden is reported according to SDI and World Bank regions.^5^ Additionally, we list countries with the highest burden and prevalence rates for orofacial clefts.

## Results

### Global Burden

**Figure 1.**
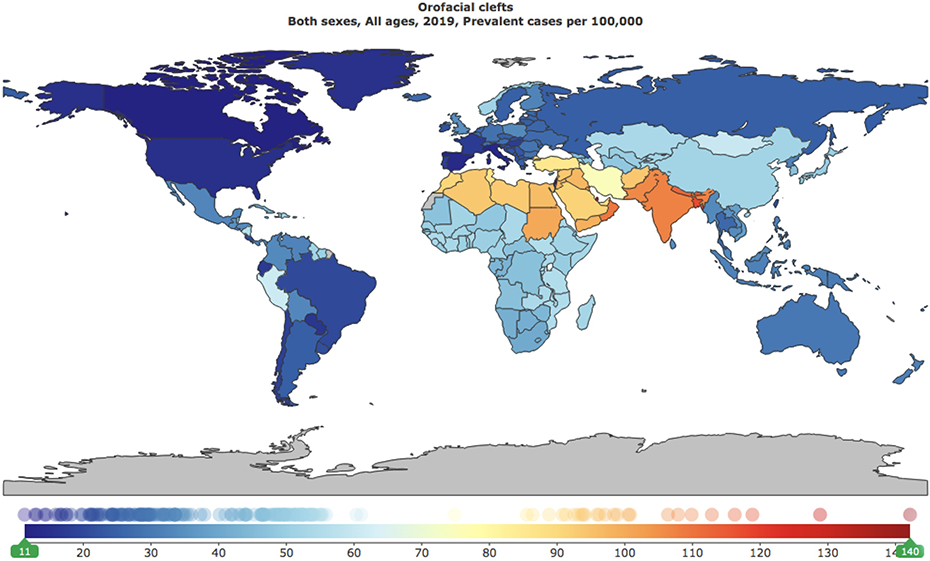
Global prevalence rates of orofacial clefts per 100,000 individuals, all ages, both sexes, 2019.

**Figure 2.**
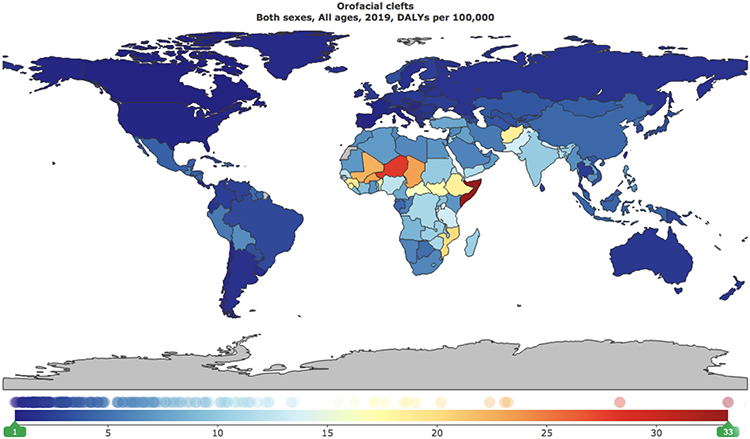
Global burden rates of orofacial clefts per 100,000 individuals, all ages, both sexes, 2019.

In 2019, the global burden of orofacial clefts was estimated to be 529,758.92 DALYs (95% UI: 362,492.88 – 798,419.69 DALYs), while the global prevalence of orofacial clefts was estimated to be 4.6 million (95% UI: 3.8 – 5.7 million). (Figure 1 and Figure 2)

The global rate per 100,000 of DALYs was estimated to be 6.85 (95% UI: 4.68 – 10.32). The highest burden of orofacial clefts was noted in Sub-Saharan Africa (13.11 DALYs per 100,000; 95% UI: 6.26 – 28.42 DALYs per 100,000), followed by South Asia (10.84 DALYs per 100,000; 95% UI: 6.87 – 16.33 DALYs per 100,000), Middle East/North Africa (6.81 DALYs per 100,000: 95% UI: 4.59 – 9.61 DALYs per 100,000), East Asia/Pacific (4.37 DALYs per 100,000; 95% UI: 3.28 – 5.84 DALYs per 100,000), Latin American/Caribbean (3.26 DALYs per 100,000; 95% UI: 2.47 – 4.26 DALYs per 100,000), Europe/Central Asia (2.37 DALYs per 100,000; 95% UI: 1.6 – 3.32 DALYs per 100,000), and North America (0.96 DALYs per 100,000; 95% UI: 0.61 – 1.39 DALYs per 100,000). (Supplemental Table 1)

There was a negative correlation between disease burden and SDI. The highest burden of orofacial clefts was reported in low SDI countries (15.06 DALYs per 100,000; 95% UI: 7.84 – 30.75 DALYs per 100,000), followed by low-middle SDI countries (9.18 DALYs per 100,000; 95% UI: 6.27 – 13.67 DALYs per 100,000), middle SDI countries (5.21 DALYs per 100,000; 95% UI: 3.76 – 6.99 DALYs per 100,000), high-middle SDI countries (3.69 DALYs per 100,000; 95% UI: 2.66 – 5.04 DALYs per 100,000), and high SDI countries (1.97 DALYs per 100,000; 95% UI: 1.25 – 2.90 DALYs per 100,000). A gender-stratified analysis of global burden of orofacial clefts per geographic area and SDI is also included in Supplemental Table 1.

### Global Prevalence

The global prevalence rate per 100,000 was estimated to be 59.68 (95% UI: 48.63 – 73.32 per 100,000). The highest prevalence of orofacial clefts was noted in South Asia (107.55 per 100,000; 95% UI: 86.98 – 133.27 per 100,000), followed by Middle East/North Africa (90.10 per 100,000; 95% UI: 73.09 – 110.72 per 100,000), Sub-Saharan Africa (52.15 per 100,000; 95% UI: 42.59 – 63.97 per 100,000), East Asia/Pacific (45.47 per 100,000; 95% UI: 37.12 – 55.51 per 100,000), Europe/Central Asia (32.40 per 100,000; 95% UI: 26.22 – 39.72 per 100,000), Latin America/Caribbean (29.62 per 100,000; 95% UI: 24.32 – 36.05 per 100,000), and North America (13.98 per 100,000; 95% UI: 11.29 – 17.15 per 100,000). The highest prevalence of orofacial clefts was noted in low-middle SDI countries (83.12 per 100,000; 95% UI: 67.47 – 102.47 per 100,000), followed by low SDI countries (71.22 per 100,000; 95% UI: 57.88 – 87.52 per 100,000), middle SDI countries (58.34 per 100,000; 95% UI: 47.67 – 71.56 per 100,000), high-middle SDI countries (44.62 per 100,000; 36.23 – 54.83 per 100,000), and high SDI countries (30.51 per 100,000; 95% UI: 24.85 – 36.96 per 100,000). A gender- stratified analysis of global prevalence of orofacial clefts per geographic area and SDI is also included in Supplemental Table 1.

### Burden and Prevalence Stratified by Country

We also analyzed the global burden and prevalence of orofacial clefts per country. The highest burden of orofacial clefts was noted in Somalia (33.27 DALYs per 100,000; 95% UI: 6.64 – 140.75 DALYs per 100,000), followed by Niger (28.33 DALYs per 100,000; 95% UI: 5.89 – 106.62 DALYs per 100,000), Chad (23.23 DALYs per 100,000; 95% UI: 6.26 – 79.91 DALYs per 100,000), Burkina Faso (23.08 DALYs per 100,000; 95% UI: 6.17 – 79.99 DALYs per 100,000), Mali (22.40 DALYs per 100,000; 95% UI: 5.95 – 75.15 DALYs per 100,000), Mozambique (20.17 DALYs per 100,000; 95% UI: 6.94 – 56.62 DALYs per 100,000), Guinea (19.03 DALYs per 100,000; 95% UI: 6.46 – 53.06 DALYs per 100,000), Afghanistan (18.64 DALYs per 100,000; 95% UI: 8.88 – 45.21 DALYs per 100,000), Ethiopia (18.49 DALYs per 100,000; 95% UI: 6.20 – 48.92 DALYs per 100,000), Sierra Leone (17.64 DALYs per 100,000; 95% UI: 6.07 – 48.15 DALYs per 100,000), Benin (17.21 DALYs per 100,000; 95% UI: 6.38 – 46.80 DALYs per 100,000), South Sudan (17.10 DALYs per 100,000; 95% UI: 6.34 – 45.52 DALYs per 100,000), Central Africa (16.23 DALYs per 100,000; 95% UI: 6.08 – 42.71 DALYs per 100,000), Burundi (15.49 DALYs per 100,000; 95% UI: 5.90 – 45.91 DALYs per 100,000), and Pakistan (13.51 DALYs per 100,000; 95% UI: 7.54 – 23.78 DALYs per 100,000). (Supplemental Table 1)

The highest prevalence of orofacial clefts was noted in Palestine (142.15 per 100,000; 95% UI: 115.46 – 174.51 per 100,000), followed by Qatar (128.85 per 100,000; 95% UI: 102.42 – 159.03 per 100,000), Bangladesh (118.85 per 100,000; 95% UI: 94.54 – 147.68 per 100,000), Bhutan (116.25 per 100,000; 95% UI: 92.98 – 145.47 per 100,000), Nepal (112.86 per 100,000; 95% UI: 90.49 – 139.64 per 100,000), Oman (109.88 per 100,000; 95% UI: 87.93 – 134.31 per 100,000), India (107.88 per 100,000; 95% UI: 87.21 – 133.64 per 100,000), Pakistan (106.39 per 100,000; 95% UI: 85.28 – 131.43 per 100,000), Kuwait (100.78 per 100,000; 95% UI: 80.02 – 124.90 per 100,000), Sudan (100.53 per 100,000; 95% UI: 81.30 – 124.74 per 100,000), Yemen (98.87 per 100,000; 95% UI: 79.12 – 120.64 per 100,000), Jordan (98.39 per 100,000; 95% UI: 78.63 – 122.64 per 100,000), Iraq (97.10 per 100,000; 95% UI: 78.03 – 120.01 per 100,000), Egypt (96.21 per 100,000; 95% UI: 77.19 – 118.66 per 100,000), and Lebanon (95.89 per 100,000; 95% UI: 76.78 – 118.37 per 100,000). (Supplemental Table 1).

## Discussion

Clefts of the lip and palate are the most common orofacial congenital anomalies.^3^ Despite guidelines strongly recommending that clefts of the lip should be repaired within the first year of life and that clefts of the palate should be treated by 18 months of age, a significant backlog of untreated patients around the globe persists.^1,3^ Importantly, untreated clefts of the lip and/or palate have been associated with increased patient morbidity and mortality.^2^ Consequently, a significant number of international cleft care organizations have attempted to address this persisting backlog of untreated patients through global initiatives that have varied considerably in scope, sustainability, longevity, geographical distribution, and model of cleft care delivery.^4^ Recently, Massenburg et al. have demonstrated that there is a significant strong negative association between the global burden of orofacial clefts and the surgical workforce, suggesting that reinforcing the global surgical workforce in geographical areas that suffer from a high burden of orofacial clefts may aid in addressing the backlog of untreated patients in those areas.^3^

In this manuscript, we evaluated updated data from the GBD 2019 study to assess the global prevalence and burden of orofacial clefts with the hope that this updated analysis may serve as a guide for organizations and healthcare providers engaged in global cleft care delivery. This analysis offers the most up to date estimates of the global burden and prevalence of orofacial clefts compared to previous analysis.^3^ These results underline global burden and prevalence of orofacial clefts, as well as prevalence and burden stratified by country, World Bank region, and SDI, while highlighting countries with the highest prevalence and burden rates. The goal of this analysis was to assist countries, international organizations, funding agencies, and policy makers involved in global cleft care to plan initiatives and channel resources accordingly towards countries and regions carrying the highest prevalence and burden of congential orofacial clefts.

Our updated analysis of the GBD 2019 study demonstrates that the estimated global prevalence (4.6 million; 95% UI: 3.8 – 5.7 million vs. 10.8 million; 95% UI: 9.9 – 11.7 million), prevalence rate (59.68 per 100,000; 95% UI: 48.63 – 73.32 per 100,000 vs. 141.56 per 100,000; 95% UI: 130.17 – 152.53 per 100,000), burden (529,758.92 DALYs; 95% UI: 362,492.88 – 798,419.69 DALYs vs. 652,084 DALYs; 95% UI: 411,089 – 1,107,193 DALYs), and burden rate (6.85 DALYs per 100,000; 95% UI: 4.68 – 10.32 DALYs per 100,000) have decreased since 2017. This suggests that current strategies in place to address the global backlog of untreated patients with orofacial clefts have been effective to some extent.^3^ This is also consistent with the previously observed trend that the global burden of orofacial clefts has decreased significantly over the last few decades.^3^ Nevertheless, our manuscript redemonstrates that geographical and socioeconomic disparities in the global burden and prevalence of orofacial clefts persist. We show that there is an inverse relationship between SDI and burden of orofacial clefts, and that Sub-Saharan Africa, Middle East/North Africa, and South Asia are the regions carrying the most significant burden of orofacial clefts. Addressing the persistent burden of orofacial clefts in these regions is particularly challenging given that a significant number of countries in them are impacted by a combination of low SDI, lack of appropriate resources and infrastructure, in addition to ongoing geopolitical instability and conflicts.^3^ These findings highlight that organizations and individuals that are heavily engaged in global cleft care delivery should focus their initiatives and resources on these countries and regions. This should be done in a collaborative fashion including the local authorities and governments in order to better understand challenges facing the local population, the context in which the burden of orofacial clefts persists, and to ensure that efforts translate into longitudinal, sustainable initiatives.

A major limitation of this manuscript is our inability to determine through the GBD study methodology details regarding the orofacial clefts. This prevents us from stratifying our analysis by type and severity of cleft. Future efforts will focus on performing a stratified analysis of different cleft conditions, temporal variations over the last several decades, as well as their psychosocial and speech outcomes on patients. Additionally, we do not necessarily capture all the burden and sequelae associated with orofacial clefts, some of which may persist even after surgical correction. This includes psychosocial considerations, as well as dental, nutritional, speech, and other craniofacial considerations. Moreover, data in countries with low SDI and regions that carried most of the burden of orofacial clefts typically lack comprehensive healthcare data which renders generating more accurate estimates of disease burden and prevalence more challenging. Another limitation of our manuscript is that we report national average rates, which can potentially mask underlying variations between states and different geographic locations and fail to highlight areas that are more affected by the disease. Nevertheless, this manuscript supplements previously published data from the GBD study and provides the most current estimates of global prevalence and burden of orofacial clefts.

## Conclusion

Global geographic, socioeconomic and demographic disparities in burden and prevalence of orofacial clefts persist despite a global decrease over the last few decades. This manuscript supplements existing data on orofacial clefts and provides the most up-to-date estimates on the global burden and prevalence of orofacial clefts, their regional and sociodemographic distributions, as well as the countries that are most afflicted by this condition. We believe the data we provide can guide and direct future investments, resources, and initiatives from individuals and organizations invested in global cleft care delivery, with the goal of building sustainable cleft care capacity where it is needed the most.

## Supplementary Material

Tables Supplemental (SDC) **Supplemental Table 1.** Global burden and prevalence rates of orofacial clefts, 2019.
