# Supplementary material for: Global Prevalence and Burden of Orofacial Clefts: A Systematic Analysis for The Global Burden of Disease Study 2019
Source: J Craniofac Surg. Author manuscript; Available in PMC 2023 Oct 23. (PMC10592431; doi:10.1097/SCS.0000000000009591)
Supplement: Tables Supplemental (SDC) — Supplemental Table 1. Global burden and prevalence rates of orofacial clefts, 2019. [file NIHMS1917803-supplement-Tables_Supplemental__SDC_.docx]

| **Supplemental Table 1. Global Burden and Prevalence Rates of Orofacial Clefts, 2019.** | | | | | | |
| --- | --- | --- | --- | --- | --- | --- |
|  | **2019 DALYs per 100,000 Population (UI)** | | | **2019 Prevalence per 100,000 Population (UI)** | | |
| ***Region*** | **All** | **Male** | **Female** | **All** | **Male** | **Female** |
| ***Global*** | 6.85  (4.68 – 10.32) | 7.04  (4.70 – 11.97) | 6.66  (4.36 – 11.02) | 59.68  (48.63 – 73.32) | 62.09  (50 .67 – 76.26) | 57.25  (46.47 – 70.49) |
| ***East Asia/Pacific*** | 4.37  (3.28 – 5.84) | 4.32  (3.15 – 5.74) | 4.42  (3.28 – 5.99) | 45.47  (37.12 – 55.51) | 42.89  (34.87 – 52.76) | 48.09  (39.32 – 58.54) |
| ***South Asia*** | 10.84  (6.87 – 16.33) | 11.34  (6.40 – 17.69) | 10.31  (6.56 – 15.99) | 107.55  (86.98 – 133.27) | 112.35  (90.62 – 139.29) | 102.55  (82.51 – 127.30) |
| ***Europe/Central Asia*** | 2.37  (1.60 – 3.32) | 2.56  (1.73 – 3.60) | 2.19  (1.42 – 3.10) | 32.40  (26.22 – 39.72) | 33.87  (27.49 – 41.41) | 31.01  (25.07 – 37.72) |
| ***Middle East/North Africa*** | 6.81  (4.59 – 9.61) | 6.88  (4.45 – 10.03) | 6.73  (4.66 – 9.40) | 90.10  (73.09 – 110.72) | 100.57  (81.27 – 123.26) | 78.61  (63.04 – 96.91) |
| ***Sub-Saharan Africa*** | 13.11  (6.26 – 28.42) | 13.11  (6.20 – 34.29) | 13.11  (5.78 – 37.00) | 52.15  (42.59 – 63.97) | 55.64  (45.52 – 68.33) | 48.76  (39.64 – 59.75) |
| ***Latin America/Caribbean*** | 3.26  (2.47 – 4.26) | 3.60  (2.70 – 4.69) | 2.94  (2.21 – 3.93) | 29.62  (24.32 – 36.05) | 32.05  (26.39 – 38.98) | 27.29  (22.37 – 33.19) |
| ***North America*** | 0.96  (0.61 – 1.39) | 1.03  (0.67 – 1.51) | 0.88  (0.56 – 1.29) | 13.98  (11.29 – 17.15) | 15.00  (12.12 – 18.40) | 13.00  (10.47 – 15.95) |
| ***Low SDI*** | 15.06  (7.84 – 30.75) | 14.96  (7.54 – 37.46) | 15.15  (7.30 – 39.16) | 71.22  (57.88 – 87.52) | 75.83  (61.58 – 93.02) | 66.58  (53.97 – 81.60) |
| ***Low-Middle SDI*** | 9.18  (6.27 – 13.67) | 9.67  (5.89 – 14.92) | 8.68  (5.86 – 13.20) | 83.12  (67.47 – 102.47) | 87.50  (70.86 – 107.92) | 78.69  (63.46 – 96.74) |
| ***Middle SDI*** | 5.21  (3.76 – 6.99) | 5.40  (3.79 – 7.33) | 5.02  (3.64 – 6.83) | 58.34  (47.67 – 71.56) | 59.79  (48.62 – 73.32) | 56.88  (46.48 – 69.62) |
| ***High-Middle SDI*** | 3.69  (2.66 – 5.04) | 3.80  (2.72 – 5.15) | 3.59  (2.56 – 4.97) | 44.62  (36.23 – 54.83) | 45.36  (36.76 – 55.76) | 43.87  (35.58 – 54.11) |
| ***High SDI*** | 1.97  (1.25 – 2.90) | 2.05  (1.30 – 2.99) | 1.90  (1.20 – 2.78) | 30.51  (24.85 – 36.96) | 31.35  (25.54 – 37.82) | 29.67  (24.03 – 36.13) |
| ***Countries with Highest Rates of DALYs and Prevalence per 100,000 Population*** | **Somalia:** 33.27 (6.64 – 140.75) | | | **Palestine:** 142.15 (115.46 – 174.51) | | |
|  | **Niger:** 28.33 (5.89 – 106.62) | | | **Qatar:** 128.85 (102.42 – 159.03) | | |
|  | **Chad:** 23.23 (6.26 – 79.91) | | | **Bangladesh:** 118.85 (94.54 – 147.68) | | |
|  | **Burkina Faso:** 23.08 (6.17 – 79.99) | | | **Bhutan:** 116.25 (92.98 – 145.47) | | |
|  | **Mali:** 22.40 (5.95 – 75.15) | | | **Nepal:** 112.86 (90.49 – 139.64) | | |
|  | **Mozambique:** 20.17 (6.94 – 56.62) | | | **Oman:** 109.88 (87.93 – 134.31) | | |
|  | **Guinea:** 19.03 (6.46 – 53.06) | | | **India:** 107.88 (87.21 – 133.64) | | |
|  | **Afghanistan:** 18.64 (8.88 – 45.21) | | | **Pakistan:** 106.39 (85.28 – 131.43) | | |
|  | **Ethiopia:** 18.49 (6.20 – 48.92) | | | **Kuwait:** 100.78 (80.02 – 124.90) | | |
|  | **Sierra Leone:** 17.64 (6.07 - 48.15) | | | **Sudan:** 100.53 (81.30 – 124.74) | | |
|  | **Benin:** 17.21 (6.38 – 46.80) | | | **Yemen:** 98.87 (79.12 – 120.64) | | |
|  | **South Sudan:** 17.10 (6.34 – 45.52) | | | **Jordan:** 98.39 (78.63 – 122.64) | | |
|  | **Central Africa:** 16.23 (6.08 – 42.71) | | | **Iraq:** 97.10 (78.03 – 120.01) | | |
|  | **Burundi:** 15.49 (5.90 – 45.91) | | | **Egypt:** 96.21 (77.19 – 118.66) | | |
|  | **Pakistan:** 13.51 (7.54 – 23.78) | | | **Lebanon:** 95.89 (76.78 – 118.37) | | |

DALYs: disability-adjusted life-years; UI: uncertainty interval; SDI: Socio-Demographic Index
